# Impact of internet usage on consumer impulsive buying behavior of agriculture products: Moderating role of personality traits and emotional intelligence

**DOI:** 10.3389/fpsyg.2022.951103

**Published:** 2022-08-29

**Authors:** Wei Jie, Petra Poulova, Syed Arslan Haider, Rohana Binti Sham

**Affiliations:** ^1^Graduate Business School, UCSI University, Kuala Lumpur, Malaysia; ^2^Department of Informatics and Quantitative Methods, Faculty of Informatics and Management, University of Hradec Králové, Hradec Králové, Czechia; ^3^Department of Management, Sunway University Business School, Sunway University, Subang Jaya, Malaysia

**Keywords:** internet usage, consumer impulsive buying behavior, personality traits, emotional intelligence, agriculture products

## Abstract

E-commerce has led to a significant increase in internet purchases. The marketing sector is very competitive these days, and marketers have a difficult task: understanding the behavior of their customers. Strategic marketing planning relies heavily on consumer behavior since the consumer acts as the user, buyer, and payer in that process. Consumers’ behavior changes in response to shifts in the factors that influence it. The purpose of this research is to show how Internet usage influence on consumer impulsive buying behavior of agriculture products through moderating role personality traits and emotional intelligence in China organic market. The data gathered in three months from January to March 2022, due to COVID-19 pandemic data was gathered through an online survey questionnaire sent by Chinese social media platforms including WeChat and an email address. The PLS-SEM technique and the SmartPLS software version 3.2.8 were used for data analyses. The result revealed that internet usage positively and significantly influences consumer impulsive buying behavior. Also, both moderator personality trait and emotional intelligence positively and significantly moderate the relationship between internet usage and consumer impulsive buying behavior. Lastly, theoretical and practical implications, and future directions were discussed.

## Introduction

Due to the fast growth of the Internet and e-commerce over the last almost two decades, online purchasing has become an increasingly essential channel for consumers to acquire agricultural goods (Richards and Rickard, 2020). According to the 2014–2015 China Agricultural Products E-Commerce Development Report, agricultural product sales on Alibaba’s e-commerce platform increased from ¥3.7 billion in 2010 to over ¥80 billion in 2014 (Huang et al., 2022), achieving an average annual growth rate of 115.64% over the past five years and demonstrating the strong momentum of online agricultural product purchases (Li, 2022). Despite the significant rise in online agricultural product purchases, China’s online agricultural product buying system is still in its infancy (Liu and Walsh, 2019). It is hampered by various causes, such as the immature online buying sector and logistical and security concerns, which has led to a reluctant and watchful attitude among many buyers (Wang et al., 2022). Numerous agricultural firms are unable to properly comprehend the aspects that impact customers’ desire to acquire agricultural goods online, resulting in the lack of relevant and effective marketing tools and approaches (Al Amin et al., 2020; Liu and Wang, 2022). Despite this, the majority of current research focuses mostly on general commerce such as apparel, books, digital products, etc., demonstrating a lack of interest in agricultural products (Truong and Truong, 2022; Zhang, 2022). Therefore, there is an immediate need to research the internet purchasing patterns of agricultural product customers (Richards and Rickard, 2020). This research vacuum will be filled by the present study, which will investigate the variables that impact the propensity to buy agricultural goods online.

Year 2020 was an exceptional year because of a pandemic. In the words of the World Health Organization (WHO), the COVID-19 pandemic “presents an unprecedented threat to public health, food systems, and the world of work.” Extreme poverty, job losses, trade and border closures, and incarceration were just a few examples of how the pandemic had a devastating impact on the economy, as well as the lives of millions of individuals. They contend that COVID-19 caused significant psychological, social and professional changes such as physical and mental health difficulties, job losses and poor savings as well as anxiety and tension during out-of-town travels (Naeem, 2020). This crisis has profoundly impacted people and their lives in many ways. All of these factors were influenced by the various constraints imposed by the governments and authorities on people’s lives. People were unable to leave their houses because of limitations on mobility and lockdowns, thus they were confined to their homes. They were able to stay in touch with family, friends, classmates from school, and even former professors and coworkers thanks to social media. They also provided a way for individuals to have fun, occupy themselves, and enjoy their auxiliary time. COVID-19’s social media channels have also played an important part in disseminating information and have been a rich and beneficial resource for COVID-19 information.

Consumer behavior has been influenced by the outbreak as well. “The period of contagion, self-isolation and economic uncertainty have altered the way consumers behave,” according to a McKinsey and Company study. These new consumer behaviors include everything from how people work, learn, communicate, travel and shop and consume to how they live at home and how they deal with health and well-being (Kohli et al., 2020). A PwC (a worldwide network of organizations supplying assurance, tax, and consulting services) poll found that COVID-19 quickly impacted consumer behavior, such as customers purchasing more basics (non-perishable grocery, housekeeping and cleaning goods, frozen meals, etc.) and using the internet shopping. Online shopping saw a significant spike in popularity during the epidemic. Because of the rise of online shopping, many businesses have turned to promotional activities on social media platforms to raise awareness of their brands and boost sales by enticing customers to share information and invite online friends (Lv et al., 2020; Haider and Tehseen, 2022). E-commerce turnover in Italy increased by 20% in the first quarter of 2020, according to Sabanoglu (2020). “Italy’s e-commerce sales climbed significantly during February and March 2020 compared to the same period of 2019. The coronavirus epidemic had a significant effect on online sales, especially over the weekend (COVID-19). Thus, the adoption of the contemporary technology tools using internet has shown a significant importance in changing consumer behavior in China (Ali Taha et al., 2021).

Impulsive buying drives consumers to purchase goods without hesitation (Kacen and Lee, 2002). The focus of impulsive buying behavior research has been on both external and internal causes. Consumers’ impulsive emotions are aided by external variables such as shopping festivals (Habib and Qayyum, 2018), the quality of shopping websites, and so on. Internal aspects always relate to personal situations and qualities, rather than being impacted by organized facilities and packaged items. Consumer buying behavior is defined as all psychological, social, and physical conduct of potential consumers as they become aware of, assess, purchase, consume, and tell other people about items and services (Lee and Yi, 2008). The impulsiveness of a customer is a unique type of consumer buying behavior. It is known as impulsive purchasing.

According to the Global Mobile Consumer Survey, 96% of Chinese people own a cellphone, making it a vital part of their lives. 93% of waking users check their phones in less than an hour. The generation born in the late 1980s and after is the primary consumer group; they spend more time on Internet usage and are thus more likely to be influenced by WeChat moments with friends and other social media “opinion leaders.” About 40% of internet purchases are impulsive. Although China has the fastest expanding organic sector, customer views about organic food items *via* social commerce are less well understood. Nowadays, customers are rather active in social groups in which they communicate with others to get other people’s ideas and comments on items, and then evaluate those products to understand how well they perform. China’s fast socioeconomic growth has been aided by the modernization and industrialization of agricultural food production. Chinese agriculture is increasingly reliant on modern agricultural pesticides, posing increased health and environmental risks (Zhang, 2022). Food manufacturing has been shown to include dangerous quantities of chemicals and food safety issues (Akbar et al., 2019). By taking into account the generally established significance of personality in determining consumer behavior, this study will examine that internet usage has utmost effect on consumer impulsive buying behavior with moderating effect of personality trait and emotional intelligence.

The word “personality” comes from the Latin word “persona,” which means “to speak through.” This Latin phrase refers to the theatrical masks used by performers in ancient Greece and Rome. As a result, personality is employed to influence people through their exterior appearance. However, only the outside appearance. Although significant for personality traits, it does not make up the entire personality. Gangai and Agrawal (2016) define personality traits as “relatively persistent qualities of individuals that exhibit consistency across their lifetimes and across a wide range of settings.” The current research looks at the link between personality factors and Internet usage. Because there are so many different personality qualities to pick from in the larger psychological literature, it’s crucial to think about what personality traits to explore in connection to Internet usage first. Fortunately, the Big Five model is widely accepted as a unified, cost-effective conceptual framework for personality.

Impulse buying is characterized by subjective bias and quick decision-making for instant acquisition. Impulse buying behaviors accounted for a significant share of consumer purchases, therefore academics and practitioners are constantly considering such unreflective and unplanned purchases. According to Wang et al. (2022), 30–50% of retail transactions are impulsive purchases. Haider et al. (2022) stated that the online shopping environment is more conducive to impulsive spending than its physical equivalent, claiming that 40% of online consumer expenditures are the consequence of impulsive purchases. For these reasons, impulsive buying behavior provides a wide range of opportunities for academics interested in consumer behavior research, as well as practitioners interested in investigating this phenomenon because it accounts for a major portion of a company’s earnings. The theory of reasoned action (TRA) and the theory of planned behavior (TPB) and its descendants (i.e., UTAUT and TAM) are widely used theoretical frameworks under these schools of thought (Toh and Selvan, 2015), and they cover a wide range of consumer behavior and decision-making processes (Cheng and Huang, 2013).

According to Liu and Walsh (2019), impulsive online shopping has become an epidemic due to the rapid expansion of e-commerce and advances in information technology. In 2015, sales of social commerce, also known as one type of e-commerce that uses consumer engagement, customer participation, and social networks to enable online selling, totaled $5 billion, with a significant growth projected in the future (Chung et al., 2017). Consumers today are more likely to engage in hedonic and experimental consumption, such as impulsive buying, since they like shopping more than they need it. The 21st century is the era of computers and the Internet. The Internet is becoming an increasingly vital part of people’s life as time and society progress. People also have a range of instruments for using interconnectivity, like mobile phones, computers, and so on. They may access necessary knowledge and information on the Internet at any time and from any place. Students, for example, may utilize the Internet to gain access to a greater range of extracurricular information and broaden their perspectives; office employees can acquire what they need with a mouse click, and the entire globe can be viewed.

Three of the Big Five personality qualities: agreeableness, conscientiousness, and extraversion—were shown to be inversely connected to Internet usage in the current study. In other words, students who were more introverted, less pleasant, and less conscientious used the Internet more. Future studies on individual Internet usage tendencies might potentially look into a range of specialized personality qualities that are too many to list here. Furthermore, using personality traits contextualized to computer and Internet usage may be effective in expanding validity correlations in this field of research. It will be interesting to see if the influence of other conceptions and determinants on Internet usage can be measured after personality traits have been taken into consideration.

## Literature review

### Supporting theory

Theory of planned behavior predicted that behavioral control and subjective norms would be the means of influencing one’s attitude and behavior (Toh and Selvan, 2015). The three components of TPB are attitude formation, perceived behavioral control, and subjective norms (Yang et al., 2018). Because of this, one’s actions and attitude may be anticipated. According to this theory, people’s intentions to carry out a given behavior are influenced by their attitude (i.e., their attitude toward organic food purchasing in this research), their perception of behavioral control, and their subjective norms (i.e., the value of other people’s views) (Cheng and Huang, 2013). Consumer attitudes regarding organic food have been studied using this idea. It is via the preservation of social norms that the impact of other people’s social pressure on adopting or not adopting a certain behavior is maintained. If you’re not careful, these societal influences might have a greater impact on your behavior than you realize. Both the United Kingdom’s and Greece’s intentions to purchase environmentally friendly items have been noted by Yang et al. (2018). Social standards and environmental awareness, have forced environmental conservation (Brandão and da Costa, 2021). Pro-environmental behavior has been shaped by society and personal values. Purchasing environmentally friendly items was found to be influenced by these considerations. Kim and Chung (2011) used this TPB to analyze the purchasing choices of organic personal care items and discovered that other people’s experiences with organic goods had an impact. Studies on students in China, the United States, and South Korea have shown that organic apparel purchases were made based on personal significance. Peer and social effect on organic product purchases was shown to be considerable by Pandey and Khare (2017). They used the TPB to describe their organic purchasing selections.

### Consumer impulsive buying behavior

Impulsive buying behavior is defined as a spontaneous response to a stimulus that results in a persistent desire to purchase a certain product or brand despite having no prior intention or necessity to do so (Badgaiyan and Verma, 2014). It is also not seen as a reminder or a habitual response. This sudden and overwhelming desire to acquire has been linked to a variety of causes that may be divided into two categories: individual-driven factors and market-driven ones (Tendai and Crispen, 2009). The first looked into consumer psychology and how it influences behavior. Shanks et al. (2010) define impulse buying as “a quick, hedonically complicated purchase behavior in which the speed of the impulsive purchase prevents any careful, intentional assessment of alternative or future ramifications.” In line with the aforementioned definitions, Truong and Truong (2022) have supplied a more detailed description, indicating that impulsive purchasing is defined as a one-time purchase made without any prior planning to buy a certain product category or complete a specified task.

It is essential to analyze the components or factors, such as characteristics, attitudes, behaviors, and participation, in light of their influence on buyer behavior (O’leary, 2017). With study factors such as increased technology, the digital revolution, aging, income levels, lifestyle changes, environmental concern, etc. influencing various components, consumer behaviors vary. In a similar vein, prior research (Zwierzyński, 2018) found that the COVID-19 pandemic will result in considerable continuous innovations across society, with many of these inventions having the potential for long-term social effects. Digital technologies are considered to be a component of these massive systemic transformations.

Customer engagement also relies on the amount of service convenience provided by organizations; when businesses increase the level of service convenience, this influences the channel switching behavior of customers. In the context of the Omni channel retailing business, both offline search convenience and online purchase convenience influence customers’ showroom behavior (Shankar et al., 2021). In addition, these components of convenience may assist identify motivations, hurdles, personality characteristics, and the influence of culture in consumer adoption, so facilitating the identification of facilitators and inhibitors (Jain et al., 2022). A meta-analysis done by Jayawardena et al. (2022) revealed that gamification-based online engagement tactics help merchants increase consumer engagement and participation. This strategy can help improve online education, online brand engagement, and information system engagement.

Kacen and Lee (2002) published an article in Marketing and Branding Research called Effects of Personality on Impulsive Buying Behavior: Evidence from a Developing Country. Openness, Extraversion, Conscientiousness, and Neuroticism were found to have a substantial influence on Impulse Buying Behavior, but Agreeableness had no effect. Many studies on impulse buying behavior look at website-related elements and their influence on the development of impulsive buying behavior. Adelaar et al. (2003), for example, evaluated and explored the influence of several media types (video, still pictures, and text) on impulsive buying behavior. Furthermore, Wells et al. (2011) found that personal characteristics and website quality has a substantial impact on the desire to buy impulsively.

When evaluating a product, younger consumers interact with others as members of a social group and solicit their opinions. This social engagement boosts user interactions and user-generated information in several ways, such as product suggestions, customer reviews, and discussion forums. These actions enable online shoppers to make more educated and precise purchasing choices, influencing their behavior toward a particular product. According to the notion of planned behavior, individuals’ thoughts influence their intentions to behave in a certain manner (Toh and Selvan, 2015). For instance, attitude is a strong predictor of organic food purchase intent, with a considerable positive correlation between the two. According to a recent consumer study, individuals do not regard their specific purchases as incorrect and, in fact, retroactively give a good opinion of their activity. Interestingly, Rook (1987) finds that while a small percentage of informants (only 20%) report feeling “terrible” about their impulsive purchases, an amazingly significant percentage (41%) report feeling “good” about them, which is hilarious given that the definition does not allow for such conduct.

### Internet usage and consumer impulsive buying behavior

Purchasing behavior is influenced by consumers’ unique personalities; each individual behaves and purchases differently based on their own personality (Yang et al., 2018). In their quest for knowledge, the Chinese rely heavily on the Internet, which has been shown to be their preferred resource. Quality signals such as nutritional content, production process, natural and animal welfare, shopping experiences, and online reviews help to shape customer attitudes that influence purchase decisions. E-commerce activities have pushed people to participate in online impulse purchasing, which is an unplanned purchase based on strong feelings for a product (Lim et al., 2017). Decisions on organic food purchases are also influenced by subjective and personal norms.

There is evidence that social commerce use is increased by peer consumers’ information sharing, rating, recommendations, reviews, and referrals (Huang et al., 2022). Marketing firms use the Internet to produce and disseminate important information since it enables customers with similar interests to communicate with one other. The benefit of organic food may be shown via these personal networks, and the consumption of organic food can be increased, as well as consumer loyalty increased. As China’s better internet technology has expanded internet users and the mobile internet has revolutionized consumption patterns and consumer attitudes, the social shopping phenomena reflects this. Consumers in China are becoming more health-conscious and conscientious about their eating habits, such as include more whole grains and vegetables in their diets. Genetically modified organisms (GMOs) and synthetic chemicals (such as fertilizers) are not permitted in organic food. As a consequence, internet usage has a positive impact on customers’ impulsive purchasing habits.

H1: Internet usage has a positive influence on consumer impulsive buying behavior.

### Personality traits as moderator

Individual buying behaviors vary; each consumer acts and consumes differently depending on his or her personality (Kacen and Lee, 2002). Some people are interested in how societal well-being influences their diet and health choices. Impulsive buying is an unexpected purchase that occurs as a result of comparing alternative purchase intents with actual outcomes. Consumers can communicate product comments and additional information through information sharing, suggestions, and referrals (Liu and Walsh, 2019). Furthermore, the internet is the most common method via which Chinese individuals search for information. The internet, in particular, plays an important role in Chinese organic consumption, and prior buying experiences of organic product customers may be found online through reviews. The number of product reviews can have a big impact on new customers’ purchase decisions since it boosts their confidence in making a selection.

Individual features that may be utilized to separate two people are referred to as traits. When the preceding literature analysis is taken into account, it is feasible to conclude that the impulsive purchasing study is focused on customer characteristics. A trait, according to Ajzen (2005), is a cross-situational individual difference that is temporally stable. It describes the traits of individuals that may be utilized to differentiate two people. The investigation of personality traits may be seen in the literature on impulsive purchase behavior (Gangai and Agrawal, 2016). Irrationality has long been regarded as a personality feature in psychological theory and study (Brandão and da Costa, 2021). McCrae and Costa Big Five model is recognized as one of the most important benchmarks in the trait theory of personality. Individuals can display all five aspects, although they may score well in one or more of them while scoring poorly in others. Extraversion, agreeableness, conscientiousness, neuroticism, and openness to experience are the Big Five Dimensions.

Extraversion describes an extroverted, extremely sociable, forceful, energetic, and thrill-seeking personality. Extraverts are drawn to interpersonal relationships. Openness to experience is associated with being creative, curious, and imaginative, as well as being unusual. They seek out new experiences and like experimenting with new concepts. Low-scoring individuals can be described as traditional and unanalytical, with a propensity for the known and routine. Agreeable individuals are usually trustworthy, forgiving, kind, warm, cooperative, and selfless. They regard interpersonal ties highly. Achievement, hard effort, responsibility, and reliability are all traits associated with conscientiousness. They are more risk-averse and want to develop long-term relationships. Finally, neuroticism is associated with anxiety, fear, depression, and poor emotional adjustment (Sinha et al., 2014). Neurotic persons do not have a socialistic outlook and avoid circumstances that need control. Because personality traits are used to predict consumer behavior, they’ll likely have an impact on online impulse purchases. As a result, personality factors have been shown to modulate the relationship between Internet use and Consumer Impulsive Buying Behavior.

H2: Personality traits moderate the relationship between Internet usage and consumer impulsive buying behavior.

### Emotional intelligence as moderator

Impulse buying is the spontaneous, instant action of purchasing a product unsupported by any previous plan, intent or decision of owning the product. Emotions play a significant influence in all of these decisions since every purchase event is an experience for the customers involved (Park and Dhandra, 2017; Ran et al., 2021). The area between a desire to buy something and the actual purchase is where emotions, among other things, play a significant influence. The stimulation of desire, as well as the procedures that lead to the final act of purchasing the object, is reliant on the individual’s emotion management mechanisms (Habib and Qayyum, 2018). Handling emotions, managing emotions, regulating emotions, and even sensing emotions all become essential factors in purchasing decisions (Soodan and Pandey, 2016). However, from the standpoint of the marketer, it is a critical instrument for provoking the client to satisfy his transitory needs. Understanding the influence of Emotional Intelligence on impulse buying behavior has been a major topic of debate.

Marketers and psychologists have differing perspectives on impulsive buying. Marketers typically believe that impulse buying occurs primarily as a result of a market impact on customers. However, the primary reason for such purchases is dictated by external variables. The emotional intelligence of a person is one of the areas of human difference (Sinha et al., 2014). Most people’s shopping experiences are defined by their affective capacity to be self-aware, recognize emotions in others, and handle emotional signals and information. Different methods for defining Emotional Intelligence have been developed, and there is controversy about how the word should be used. Darwin’s study on the need for emotional expression for survival and second adaptation may be traced back to the beginnings of emotional intelligence. Several notable academics in the intelligence field of study began to notice the relevance of non-cognitive characteristics in the 1900s, even though conventional definitions of intelligence emphasized cognitive aspects such as memory and problem-solving. Not much research has been conducted to determine how emotional intelligence impacts or influences consumer purchasing behavior. Because impulse purchasing affects all product lines and is a significant area where organizations try to focus to entice clients, it is particularly interesting to investigate whether this crucial behavioral attribute has any significance from both the individual’s and marketer’s perspectives.

**FIGURE 1:**
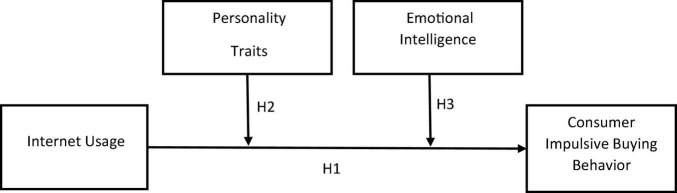
Conceptual model.

Emotion is a significant antecedent that influences decision-making. People are prone to regret, and they will go to great lengths to avoid future regrets and current regrets. Counter-factual reasoning can be used to foresee the emotional implications of hypothetical decision-making. In the context of marketing and consumer-based research, Kidwell et al. (2008) created a consumer emotional intelligence measure and concept. Amos et al. (2014) argued that the interaction of socio-demographic, situational, and dispositional characteristics might create a favorable environment for boosting impulsive buying in a meta-analysis of consumer buying behavior. Moreover, contend that psychological functioning, particularly as a type of self-regulation, might be valuable in explaining impulsive purchase behavior. Consumers frequently make unexpected and unanticipated purchases in an online setting, according to Jones et al. (2003); their intentions may be derived from the complexity or simplicity of the website. Consumer buying behavior is influenced by impulsive reactions, inadequate cognitive control, and emotions in this situation (Soodan and Pandey, 2016). As a result, EI helps to control the relationship between internet use and consumer impulse buying (see Figure 1).

H3: Emotional intelligence moderate the relationship between Internet usage and consumer impulsive buying behavior.

## Research methodology

### Sampling and population

The suggested model hypothesis was empirically examined by means of a quantitative research technique (Figure 1; Barnham, 2015). Therefore, a cross-sectional data collecting method using an online questionnaire that was most suitable for this setting was utilized to determine the structural relationship between contemplative latent components. It is not only congruent with this research, but also geographically unrestricted and has the lowest survey cost and fastest response rate. The online survey questionnaire was divided into two sections to obtain data on organic food impulsive purchases made online. The purpose of the first component of the questionnaire was to ensure that respondents had experience purchasing organic food goods online. In the first segment, a screening question with a nominal scale (yes/no) was implanted for this purpose. If the respondent had not purchased any organic food online, they were forwarded to the survey’s conclusion page. In reality, respondents who lacked organic purchasing expertise were voluntarily excluded from the research; hence, the study sample consists of experienced online purchasers of organic food. The majority of respondents bought organic milk, organic grains, organic veggies, and organic eggs. The following part was intended to capture demographic information such as age, gender, education, and income, along with Internet usage use and time spent on social media. The purpose of the last segment was to evaluate the structural relationship between latent hypotheses.

In China, the research was carried out. During the months of January–March 2022, due to COVID-19 pandemic data was gathered through an online survey questionnaire sent by Chinese social media platforms including WeChat and an email address. The questionnaire was translated from English to Chinese for this study’s Chinese participants, and both English translations were compared to identify and correct any inconsistencies to ensure the intended meaning of the questions to be answered by respondents were accurately conveyed in both translations. A total of 360 respondent were requested to fill out an online survey questionnaire, however only 285 valid replies were obtained (response rate = 79.16%) with 145 Male (49.1%) and 140 females (50.9%) responding as presented in Table 1. Females were more likely than men to use social media and choose organic food, according to the gender ratio. The majority of respondent were 20- to 30-year-old (42.8%) and 31 to 40 (42.1%) year old. The conclusions of this research are based on a large sample of well-educated individuals, with 39.3.% of those who participated holding a Bachelor degree, Masters (37.2%) or above. One-half of the samples make more than 1,500–8,000 yuan every month.

**TABLE 1:** Descriptive statistics.

Demographics	Categories	Frequency	Percent
Gender	Male	140	49.1
	Female	145	50.9
Age	20–30	122	42.8
	31–40	120	42.1
	41–50	43	15.1
Education	Bachelor	112	39.3
	Masters	106	37.2
	MS/MPhil	51	17.9
	PHD	10	3.5
	Any other	6	2.1
Experience	<1	18	6.3
	1–3	146	51.2
	4–6	73	25.6
	>6	48	16.8
Monthly income	<1,500 yuan	22	7.7
	1,500–4,000 yuan	94	33.0
	4,001–8,000 yuan	98	34.4
	>8,000 yuan	71	24.9

### Instrument

The scale developed by Deane et al. (1998), which included 8-items scale, was used to measure independent variable Internet usage. Furthermore, the 7-items scale was used for dependent variable consumer Impulsive buying behavior adopted from Karbasivar and Yarahmadi (2011). The two moderating variables were used in current study, personality traits the 44-items scale developed by John and Srivastava (1999) and emotional intelligence based on 16-items adopted from Wong and Law (2002). According to the Five-points Likert scale, responses ranged from 1 (strongly disagree), 2 (disagree), 3 (neutral), 4 (agree), and 5(strongly agree). Analysis of survey data is carried out utilizing Smart PLS and SEM (Structural Equation Model) software. This is because SEM is one of the most common approaches among marketing researchers for assessing new conceptual models with many complicated social structures, and PLS-SEM is a tool for more extensive statistical analysis. This research is acceptable for applying PLS-SEM since the complexity of the present model includes two variables as moderators, for a total of four variables. The subsequent section will include data analysis and interpretation of the findings.

## Research results

### Measurement model

The partial least squares- structural equation modeling (PLS-SEM) technique and the SmartPLS software version 3.2.8 were used in this study (Hair et al., 2017). PLS-SEM offers the benefit of being more adaptable while also having a better level of statistical strength (Hair et al., 2019). The PLS-SEM statistical analysis then proceeds to two stages: the evaluation of the measurement model and the evaluation of the structural model. Validity and reliability requirements for the measurement model must first be met before data can be used to determine relationships between variables (see Table 2). While evaluating measurement models, the reliability and validity of each concept are taken into consideration. In terms of construct reliability, the emphasis is on indicator loading values that indicate indicator reliability values and composite reliability, which displays the construct’s dependability and internal consistency (Memon et al., 2021).

**TABLE 2:** Measurement model and HTMT.

Constructs	Cronbach’s alpha	CR	AVE	CIBB	EI	IU	PT
CIBB	0.891	0.921	0.703				
EI	0.789	0.863	0.612	0.425			
IU	0.866	0.903	0.617	0.830	0.359		
PT	0.835	0.890	0.669	0.403	0.337	0.295	

AVE, average variance extracted; CR, composite reliability; IU, internet usage; CIBB, consumer impulsive buying behavior; PT, personality traits; EI, emotional intelligence.

Based on the bootstrapping method (T-tests for 5,000 subsamples), Hair et al. (2017) evaluated the statistical significance of factor loadings, weights, and path coefficients for each variable. In addition to factor loadings analyses, Cronbach’s alpha, composite reliability (CR), and average variance extracted (AVE) analyses were conducted (Memon et al., 2021). The validity of explicit indicator hypotheses may be assessed by their factor loadings, which suggest that loadings larger than 0.50 on two or more variables are substantially reflected in the hypotheses. Wong (2013) developed a strategy for excluding items with factor loadings between 0.40 and 0.70 from assessment if excluding observed variables increases AVE and CR values in reflective scales. It can be shown in Table 2 that the Internet usage (IU), consumer impulsive buying behavior (CIBB), personality traits (PT), and emotional intelligence (EI) all have CR values more than or equal to the minimum limit value of 0.70. In other words, the validity of the study concept has been proven. Meanwhile, the question of construct validity is being examined in terms of convergent and discriminant equivalence, respectively. Each of the constructs has an AVE value of 0.617 (IU), 0.703 (CIBB), 0.669 (PT),and 0.612 (EI), indicating converging validity in AVE. The convergent validity value of each construct surpasses the minimal limit value of 0.50, in other words. As with concept discriminant validity, the HTMT value for each correlation must be less than 0.85 in order to ensure heterotrait-monotrait correlation (HTMT) validity (Sarstedt et al., 2020). Table 2 reveals that HTMT values are generally recognized and have a good degree of discriminant validity, which leads us to draw the following conclusion: Thus, it may be determined that all of the data meets the requirements of the measurement model. Thus, its validity and reliability are accepted.

### Structural equation model

**FIGURE 2:**
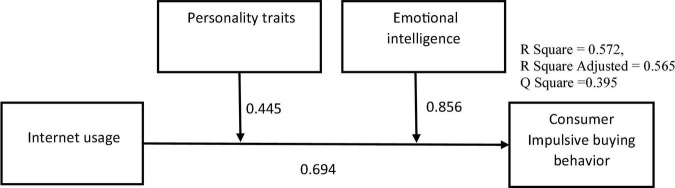
Path analyses, coefficient of determination in the PLS method.

After determining that the measurement model is adequate, the following step is to evaluate the structural model (Hair et al., 2019). In a structural model, each hypothesis is assigned a causal link, and the hypothetical relationship in a structural model is often evaluated using a route coefficient. Such as the coefficient of determination (R^2^), effect size (f^2^), and predictability relevance (Q^2^) Cohen (1998). The statistical significance of a coefficient is often determined by its t value. The *t*-value 1.65 (significance level = 10%), *t*-value 1.96 (significance level = 5%), and t-value 2.57 (significance level = 1%) are the crucial values usually employed in two-sided testing. In this research, if the hypothesis’s *t*-value (relationship between variables) was larger than the cut-off value of 1.96, then the hypothesis was validated. When the hypothesis t-value (relationship between variables) falls below the 1.96 cut-off, the hypothesis is considered invalid. Table 3 presented that internet usage has positive and significant influence on consumer impulsive buying behavior (β = 0.694, *p* < 0.001) also t-value is equal to 22.148 > 1.96. The value of β demonstrates the percentage change, illustrating that a one-unit change in internet usage results 0.694 unit change on CIBB. The results reveal that around 69.4% of the change in the dependent variable CIBB is seen, and a *p*-value < 0.001 implies a higher degree of significance, giving strong grounds for accepting hypothesis H1. The Coefficient of Determination (*R*^2^) quantifies a model’s prediction accuracy. Its value ranges from 0 to 1 and represents the cumulative effect of external latent factors on endogenous latent variables. Stronger explanatory power was indicated by higher *R*^2^ values. *R*^2^ values of 0.75, 0.50, and.25, according to Cohen (1998), are categorized as large, moderate, and low. Figure 2 illustrates the values of *R*^2^ for Consumer Impulsive buying behavior was 0.572, which is considered high and indicated that Internet usage, personality traits and emotional intelligence explained 57.2% of the variance in CIBB.

**TABLE 3:** Structural equations model results.

Hypotheses	Relationship among constructs	β	Mean	S. D.	T values	F^2^ values	*P*-values	LLCI 2.5%	ULCI 97.5%	Remarks
	**Direct effect**									
H1	IU → CIBB	0.694	0.694	0.031	22.148	0.531	0.000	0.628	0.753	Supported
	**Moderating effect**									
H2	IU × PT → CIBB	0.445	0.441	0.042	10.583	0.346	0.003	0.357	0.522	Supported
H3	IU × EI → CIBB	0.853	0.856	0.045	19.105	0.423	0.000	0.764	0.941	Supported

IU, Internet usage; CIBB, consumer impulsive buying behavior; PT, personality traits; EI, emotional intelligence; SD, standard deviation; LLCI, lower limit confidence interval; ULCI, upper limit confidence interval.

A measure of the effect size (f^2^) is the amount by which the value of *R*^2^ fluctuates when a specific exogenous construct is removed from a model to determine whether or not the exclusion has an influence on the endogenous constructs. A f^2^ value larger than 0.35 implies a considerable influence. A range between 0.15 and 0.35 suggests a moderate impact size, while a result between 0.02 and 0.15 indicates a small effect size (Cohen, 1998). According to Table 3, the independent construct Internet usage has a significant impact size on CIBB, whereas the combined constructs Internet usage (independent) and PT (moderator) have a medium effect size CIBB, Internet usage (independent) and EI (moderator) have a high effect size on CIBB. To assess the predictive significance of the inner model, Q^2^ (predictive relevance) is used. Q^2^ was calculated using the blindfold technique, whereas D was found to be 7. Utilizing a cross-validated redundancy approach, predictive significance was determined. If a value greater than zero implies predictive relevance, a value below zero suggests the model is not predictively relevant (Cohen, 1998). Figure 2 displays the Q^2^ value = 0.395 of endogenous components, which is greater than zero to illustrate the model’s predictive relevance. Furthermore, the moderating impact of personality traits on the relationship between Internet usage and CIBB is positive and significant (β = 0.445, T = 10.583, *p* < 0.05). Also, emotional intelligence positively and significantly moderates the relationship between Internet usage and CIBB (β = 0.853, T = 19.105, p < 0.05). Hence, the Hypothesis H2 and H3 were accepted.

## Discussion

This study investigated influence of internet usage on consumer impulsive buying behavior through moderating role of personality trait and emotional intelligence. The result revealed that internet usage positively and significantly influences consumer impulsive buying behavior. Also, both moderator personality trait and emotional intelligence positively and significantly moderate the relationship between internet usage and consumer impulsive buying behavior. The current study results related with prior studies; Zafar et al. (2020) indicated that consumer impulse buying has a very significant association with their emotional intelligence. Moreover, Chinese Internet usage has become the foremost source of information to gain food protection knowledge, an effective and delightful way to have more abundant product related information. Consumers often join social groups for the sole purpose of meeting their unique requirements; yet, viewing others’ expertise and experience, as well as interacting with them, has the potential to influence their attitudes regarding goods and websites. Chinese customers are wary of buying food online because they distrust food providers, therefore they go for more information for a second opinion. People with higher emotional intelligence are better able to manage the euphoric want to buy because they can cope with the temporary misery of not having the object right now (Park and Dhandra, 2017). They are not ruled by their emotions. Understanding and regulating one’s own emotions to make the best decision possible is a crucial mechanism of a highly emotionally intelligent person (Habib and Qayyum, 2018). As a result, those with higher Emotional Intelligence are less likely to feel guilty. It is critical to acquire customer trust by having items authenticated and validated by accredited institutions and the government. While technology has altered consumer behaviors, the COVID-19 epidemic has increased the rate of change. Ecommerce is a popular shopping trend that is fueled by the advantages of both traditional businesses and online platforms.

In general, emotional intelligence is thought to represent distinct differences in the ability to integrate emotional input and apply it to broader judgments. Since the (effective) operationalization of essential individual capabilities/assets is required for adapting forms, EI might well convey them, understood as either a collection of talents placed at the confluence of consciousness and feeling or as our emotional identity (Soodan and Pandey, 2016). In this sense, EI is less about feelings and more about how people successfully integrate emotions with cognition to lead to behavior, which can help to reduce adverse emotional encounters. Because, the internet can hold a large amount of data, Chinese consumers access information through online channels. As a result, the internet is the principal source of information. Consumers frequently join social groups to meet their requirements by watching others’ expertise and experiences, and these interactions have an impact on their perceptions of goods and services. Consumer impulsive buying behavior is positively related to internet usage.

## Conclusion

The Chinese government has made measures to ensure that domestic organic production meets high-quality requirements (see the General Administration of Quality Supervision, Inspection and Quarantine of the People’s Republic of China, 2012). Consumers may now buy impulsively more easily than ever before because of the Internet. Researchers should pay special attention to the enormous role that the Internet can play in making surfing more convenient and engaging, expanding the possibilities for instant satisfaction depending on mood states, and creating a hedonic buying environment. It was shown that consumer’s personality trait toward organic food had a postive and significant moderating effect on their online impulsive purchasing behavior when employing social market (Lim et al., 2017). In other words, when consumers’ sentiments toward organic food improve, online impulsive purchases likewise grow (Gangai and Agrawal, 2016). Chinese consumers get information online because the internet can store voluminous amounts of data (Liu and Wang, 2022). Internet is thus the key source of food safety information. Typically, consumers join social groups in order to meet certain requirements by examining the expertise and experiences of others, and these interactions eventually influence their opinions about goods and websites. By providing perspectives on product and vendor quality, social recommendations, ratings, and reviews have also contributed to the development of a satisfactory attitude toward organic consumption. This gives a more practical shopping experience, hence decreasing clients’ indecision throughout the purchasing choice (Truong and Truong, 2022). Indeed, Chinese consumers are cautious because they mistrust food manufacturers; as a result, they seek further information in order to evaluate alternatives. Thus, internet evaluations, reviews, and the buying experiences of others play an important part in the organic food market. This is fundamental to social trade. The results for study were consistent with findings from western research. These research’ results support the concept of a pro-environmental, organic-consumerist mentality that tries to please consumers.

Nevertheless, e-commerce is a rapidly expanding sector as a result of the COVID-19 epidemic. The findings of the present research indicate that customers are motivated if they are engaged online, which influences their behavior and attitudes. In addition, it has been reaffirmed that providing consumers with an engaging offline experience is one method to meet their demands and improve their purchasing habits. This study is essential since the business community must attract and keep consumers’ interest. Such a distinct value proposition will differentiate rivals. In exchange, the image of the brand will generate positive reinforcement. As Internet use has become the standard for almost everyone with a focus on expanded consultation and customization and content marketing to attract an audience.

## Theoretical and practical Implications

There are various ways in which this study adds to the body of information around organic food consumption. This new model for online impulsive purchases is based on consumer internet usage and includes the indirect moderating influence of personality trait and emotional intelligence in terms of media content. There has been an increase in the use of internet usage as a means of influencing purchasing decisions. However, the extent to which others have influenced has not been properly investigated. It is hoped that the findings of this study will serve as a foundation for further research on organic products sold online. Thus, a fuller knowledge of Chinese impulsive purchasing behavior is achieved by examining social attraction and media richness. This study’s theoretical underpinnings, social appeal, reinforce the importance of organic food’s influence on impulsive purchases. As a result of the rise of social commerce, consumers purchasing choices are more influenced by the opinions of others (Liu and Walsh, 2019). Using this approach, researchers may better understand how social attraction affects consumers’ willingness to purchase impulsively. The level of scientific and technological literacy among consumers might increase demand for organic foods. Consumers are more likely to choose for organic products if they have a better grasp of how they are made. Referrals and reviews, product experience, and information gleaned from online forums and groups all play a larger role in organic food buyers’ decisions than previously thought. Several key elements were found to influence organic purchasing, including precise and believable labeling, which builds confidence and a favorable attitude based on the number of organic components used, how it is cultivated and handled. Third, we looked at the influence of website personality on online impulsive purchases of organic food. The literature on social commerce websites’ technological aspects has been uneven, despite the fact that few studies have examined them. This study has added to already existing research.

Results show that managers may safely establish a relationship between a product’s identity and the identities of its customers in order to get preliminary market acceptability. Influential material such as important others, should be used by marketing managers in order to influence consumers’ purchasing decisions. As a means of influencing the value proposition of organic customers, health communities may use social media. It is possible that creating knowledge communities might be a novel strategy to realize the value of consumer expertise. The establishment of trust, mutual benefit, and a sense of belonging would be enhanced in such long-term communities. Not only does this benefit the community by increasing and preserving its collective knowledge, but it also encourages its members to take an active role in it. Health community influence may be used by entrepreneurs in order to alter their consumer value offer. Post-purchase value is derived through social engagement, exchange of opinions and evaluations by others. This may aid in the acquisition of product knowledge. Using this process, customers are able to form an emotional connection with business owners and change their attitudes. Clear consumer advantages may leave a lasting impact on the general public, resulting in increased levels of customer satisfaction and retention. People’s attitudes regarding organic food may be readily and swiftly changed by disseminating complete information through social media (Al Amin et al., 2020). People’s cognition and purchasing intents are favorably influenced by social media marketing communications, which are regarded an outstanding medium.

For decades, the effect of technology has shifted customer preferences, which has led to an explosion of e-commerce. Due to the paucity of literature in the present research area, particularly concerning the Internet’s influence on consumers’ impulsive purchasing of agriculture products with the moderating role of personality traits and emotional intelligence, the present study is quite significant for decision-makers and industry stakeholders. In addition to the COVID-19 pandemic, consumer behavior has altered over the movement control period, and customer satisfaction will continue to influence online purchase activities and decisions. Therefore, the current research needed to address the current market circumstances. In addition, this research demonstrates that customers anticipate benefits from both online and offline purchases to optimize their purchasing benefits.

Online marketers must keep their websites up to date and attractive to keep clients coming back (Lim et al., 2017). It’s a tremendous task for them to get through a tedious and infectious internet interface with only a single mouse click, Retailers that want to increase the number of impulsive purchases made on their sites should thus focus more on impulse purchasing, spending more resources in online shop design as a result. The website should be accessible and simple to use, with a nice variety of related items, such as an appetizing image for food. This will encourage impulsive purchases. Organic food websites should have more complete and rationalized web pages to attract and retain browsers with an influential interface that drives visitors to make impulsive purchases, according to the findings. As a consequence, consumers are more likely to make purchases after visiting a website that has high-quality images, appropriate typefaces, eye-catching color schemes, and detailed information about organic ingredients and farming. Consumer doubt may be lessened to some degree due to the tremendous media richness of internet items, which reduces the information asymmetry. Additionally, the answer to successful trigger of impulsive purchasing is to provide customers with appropriate exposure to the necessary sustaining stimuli with the least physical, mental, and time effort by constructing a courteous shopping experience. The research provides empirical evidence of the importance and relevance of social factors in the formation of organic attitude. According to the findings, successful websites have a strong personality that influences people’s purchase decisions.

## Limitations and future directions

In addition, several limits should be highlighted. Low external validity may be a shortcoming of the study, since participants may behave differently in real-world settings than they did during the experiment. In this study, the participants were inquisitive, but their actual purchasing behavior may alter if risks are considered more logically. We did not measure the actual amount of time spent in various areas of Internet use, which may indicate different patterns of association with personality characteristics than the percentage of overall time spent online. We were unable to determine if our measure of Internet use was range-restricted in our sample, which might have diminished the size of observed relationships. In the future, researchers will investigate the behavior of different age groups.

Due to the fact that impulse buying behavior was strongly related to emotional/affective reactions and behavior, despite the possibility that it was more likely influenced by external factors, it was somewhat challenging to distinguish between cognitive and affective impulses using survey questionnaires. If customers were aware of the phrase “purchasing,” several factors/events may have been evaluated directly. In the future, it is proposed to combine quantitative and qualitative research methodologies (such as observational or experimental research methods). Also, future study might explore how internal elements (mood and affection) impact impulsive purchases of low-involvement clothing goods, as well as impulse purchases of high-involvement products. Other characteristics, such as the perceived dangers connected with online purchasing, self-efficacy, or fear toward technology, could be included in future study. The non-random selection of participants is a significant weakness of our research. Lastly, it will be intriguing to see if the effects of other constructs and determinants on Internet use may be evaluated once personality traits have been accounted for.

Consequently, experimental studies (for instance, experimenting with different retail web-sites with different stimuli to measure stimuli and explore implications for web-page layouts) and structured quantitative studies would greatly improve our overall understanding of impulse buying phenomena in both traditional retail stores and online. Due to China’s significant usage of social media, this study’s China-based sample was suitable for analyzing the phenomena of purchasing behavior. However, the sample population may restrict generality, a problem that might be resolved by duplicating this research with samples from different nations. Comparing various generations might also be a potential future study area, considering the increasing Internet use among the elderly. This research utilizes Chinese customers on purpose. However, investigating non-users might be intriguing. Future study should construct a more realistic simulation environment in order to better observe impulsive online purchasing behavior. Future study is expected to use a bigger sample size to increase the applicability of the findings.

## Data availability statement

The raw data supporting the conclusions of this article will be made available by the authors, without undue reservation.

## Author contributions

WJ: conceptualization and writing—original draft. SH: formal analysis, software, and resources. RS: supervision, writing—review and editing, and validation. PP: conceptualization, writing—original draft, and methodology. All authors contributed to the article and approved the submitted version.
